# Even a good influenza forecasting model can benefit from internet-based nowcasts, but those benefits are limited

**DOI:** 10.1371/journal.pcbi.1006599

**Published:** 2019-02-01

**Authors:** Dave Osthus, Ashlynn R. Daughton, Reid Priedhorsky

**Affiliations:** 1 Los Alamos National Laboratory, Los Alamos, New Mexico, USA; 2 University of Colorado Boulder, Boulder, Colorado, USA; The George Washington University, UNITED STATES

## Abstract

The ability to produce timely and accurate flu forecasts in the United States can significantly impact public health. Augmenting forecasts with internet data has shown promise for improving forecast accuracy and timeliness in controlled settings, but results in practice are less convincing, as models augmented with internet data have not consistently outperformed models without internet data. In this paper, we perform a controlled experiment, taking into account data backfill, to improve clarity on the benefits and limitations of augmenting an already good flu forecasting model with internet-based nowcasts. Our results show that a good flu forecasting model can benefit from the augmentation of internet-based nowcasts in practice for all considered public health-relevant forecasting targets. The degree of forecast improvement due to nowcasting, however, is uneven across forecasting targets, with short-term forecasting targets seeing the largest improvements and seasonal targets such as the peak timing and intensity seeing relatively marginal improvements. The uneven forecasting improvements across targets hold even when “perfect” nowcasts are used. These findings suggest that further improvements to flu forecasting, particularly seasonal targets, will need to derive from other, non-nowcasting approaches.

## Author summary

It has been demonstrated in retrospective settings that flu forecasting can be improved by augmenting forecasting models with internet-based nowcasts (i.e., one-week-ahead forecasts generated with internet data). The improvement of internet-assisted forecasting models, however, has not translated to overall improvement in flu forecasting of public health-relevant targets. This is partially due to the relatively simple forecasting models that nowcasts are augmenting and partially because nowcasts do not directly inform all public health-relevant targets, such as the start, the peak timing, and the peak intensity of the flu season (i.e., seasonal targets). This paper conducts an experiment to provide clarity on the benefits and limitations of augmenting flu forecasting models with internet-based nowcasts in practice. We show that 1) nowcasts can improve flu forecasting across all geographic regions, flu seasons, and public health-relevant targets, including seasonal targets, and 2) the benefits of nowcasting are uneven, as even in the perfect nowcasting setting, only marginal improvements to forecasts of seasonal targets are possible relative to short-term forecasts. This is because nowcasts provide direct information about short-term targets but only indirect information about seasonal targets. This paper supports previous findings that nowcasts can improve flu forecasting but clarifies the limitations of those improvements, both in terms of magnitude of possible improvement as well as which targets can be improved. The continued advancement of flu forecasting hinges on the development of model improvements separate from nowcasting.

## Introduction

### Influenza forecasting background

Seasonal influenza causes substantial morbidity and mortality, including an estimated ten to fifty thousand deaths annually [1]. The ability to accurately monitor and forecast influenza outbreaks is thus of substantial public health priority. Traditional flu surveillance in the United States consists of a large network of volunteer reporting physicians and associated mortality, laboratory, and hospital surveillance, organized by the Centers for Disease Control and Prevention (CDC) [2]. This network, while expansive, has known limitations, including frequent revisions to previously issued data and a 1–2 week reporting lag. Recent work has suggested that internet data, for example search query volume or symptom reports on social media, might help fill in this gap created by the reporting lag by providing real-time estimates of sick individuals. The estimate that fills the gap is called a *nowcast*, and computing this estimate is called *nowcasting*.

Much work has been done to provide evidence that forecasting models which incorporate internet data can outperform those without. A fairly exhaustive discussion of these models is provided in our previous work [3]; some particular examples are highlighted here. In general, studies have primarily incorporated data from social media, in particular Twitter [4, 5], Wikipedia [3, 6, 7], and Google search query volume [8–10]. Initial work focused on the observation that internet data (e.g., tweet volumes) and the number of influenza infections are correlated [5]. Subsequent studies found that models including internet data perform better than autoregressive baseline models (e.g., [4, 11]). Others added internet data to compartmental models (i.e., models governed by a system of differential equations that group individuals into compartments based on their health status). Using this approach, for instance, [9] added Google Flu Trends data to a compartmental model and found that this addition allowed them to predict the peak of two outbreaks several weeks in advance. Although the vast majority of studies have focused on incorporating an individual internet data stream, some have observed that ensemble methods using multiple data streams could outperform those where only one is added [10, 11].

### The internet paradox

Though there is evidence that influenza forecasting models can be improved by incorporating internet data, it is unclear how useful these models are operationally for two reasons. First, there are a number of concerns that internet data may be unreliable, as there is no reference data to know which individuals are actually sick; untangling which traces correspond to relevant health statuses and which do not is a challenging problem. These potential data problems have now become notorious. Google Flu Trends, while initially an effective model, later substantially overestimated seasonal flu for several years in a row [12]; it no longer publishes flu estimates. Also, of the seven models that participated in the second year of the CDC’s flu forecasting challenge (four using internet data and three not), the two best performing models did not use internet data [13]. Second, previous research has typically compared relatively simple forecasting models, often autoregressive or compartmental models, to an identical forecasting model augmented with internet data (as previously mentioned). However, this approach fails to take into account the possible impact of internet data on more intricate models that arguably perform better than their more simplistic counterparts (though there has been recent work evaluating the value of nowcasting with more intricate models [14, 15]). For example, in our previous work [16], we developed the Dynamic Bayesian Model (DBM)—a flu forecasting model that does not use internet data. [16] compared DBM to all flu forecasting models that participated in the CDC’s 2015/16 and 2016/17 flu forecasting challenges, nationally, and found DBM outperformed all participating models, many of which used internet data for forecasting. These findings suggest that 1) DBM is a leading flu forecasting model and 2) internet data is not required for good flu forecasting performance.

Given all of this, it is unclear what we should conclude about internet data for flu forecasting. On one hand, there are numerous examples where internet data has improved flu forecasting in retrospective forecast settings. On the other hand, leading forecasting models make no use of internet data.

### Problem statement

In this paper, we seek to provide clarity on the value of internet data when forecasting the flu in practice. We do so by conducting a focused experiment. The questions the experiment addresses are:

1. **Can a good flu forecasting model using no internet data be improved by adding internet data?** Yes. Our experiment demonstrated that a good flu forecasting model can be improved in practice through the incorporation of internet-based nowcasts.
2. **If so, do the improvements hold across flu targets, season weeks, geographic regions, and flu seasons?** Generally yes. The model augmented with internet-based nowcasts had equal or better performance than the model without such nowcasts across all considered flu targets, geographic regions, and flu seasons. Some short-term forecasts issued in December and early January exhibited worse forecasts than the no internet model, however.
3. **What are the limitations of nowcasts, internet-based or otherwise, in terms of improved forecasting performance?** Perfect nowcasting can improve forecast accuracy for both seasonal and short-term targets. Improvements for seasonal targets, however, are much smaller than for short-term forecasts. The overall possible improvement to flu forecasting accuracy via nowcasting is limited by the uneven impact nowcasts have on forecasting various public health-relevant targets. If improvements above and beyond what can be achieved with perfect nowcasting are needed, other, non-nowcasting model improvements must be considered.

## Materials and methods

### Data

In the following sections, we describe the data sources used for the experiment: CDC’s influenza-like illness network, Wikipedia, and Google Health Trends.

#### Influenza-like illness

**Fig 1:**
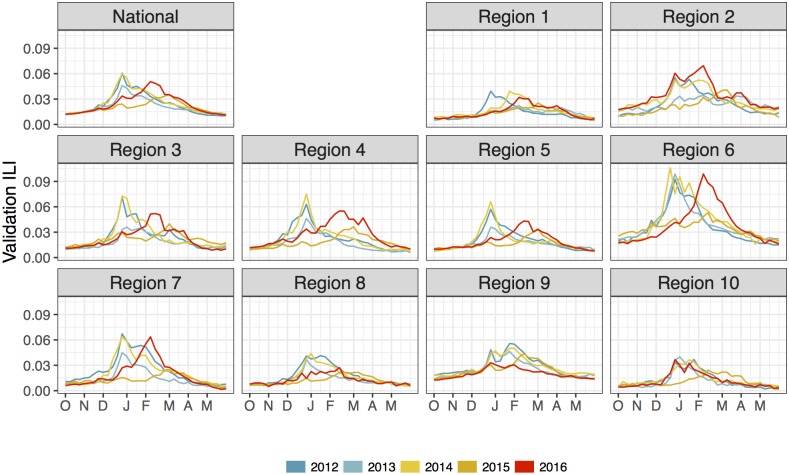
Validation ILI for the 2012 through 2016 flu seasons nationally and for all 10 HHS regions. For all regions, ILI is low at the beginning (October) and end (May) of the flu season, peaking between December and March. Different HHS regions have different ILI magnitudes, with Region 6 having more intense flu seasons, on average, than Region 1.

We use outpatient influenza-like illness (ILI) data from the 2012/13 through 2016/17 seasons for our analysis, here denoted 2012 through 2016. ILI is the number of patients that visited an influenza-like illness network (ILINet) participating clinic with symptoms consistent with the flu (temperature of 100 degrees Fahrenheit or greater and a cough and/or a sore throat with no other known cause), divided by the number of patients that visited an ILINet clinic for any reason [2]. In this paper, ILI is a proportion and refers to *weighted ILI*—a state population-weighted estimate of ILI. Fig 1 shows *validation ILI* nationally and for each of the 10 Health and Human Services regions (HHS regions), as designated by the Department of Health and Human Services, for flu seasons 2012 through 2016. Validation ILI refers to the ILI estimates issued on the *validation date*: March 2nd, 2018.

A validation date is needed because ILI estimates are perpetually updated, as the participating ILINet clinics are continuously evolving and ILI submissions are rolling. Every week when ILI estimates are issued by the CDC, previously issued ILI estimates may be modified. Changes to *initial ILI* (the ILI estimates first issued by the CDC) after the initial issue date are referred to as *backfill*. When retrospectively recreating realistic forecasting environments, ILI data available on historical forecast dates are needed. Carnegie Mellon University’s Delphi group provides access to ILI data available on historical dates (referred to as *available ILI*) through their real-time epidemiological data API [17], facilitating faithful recreations of forecasting environments.

**Fig 2:**
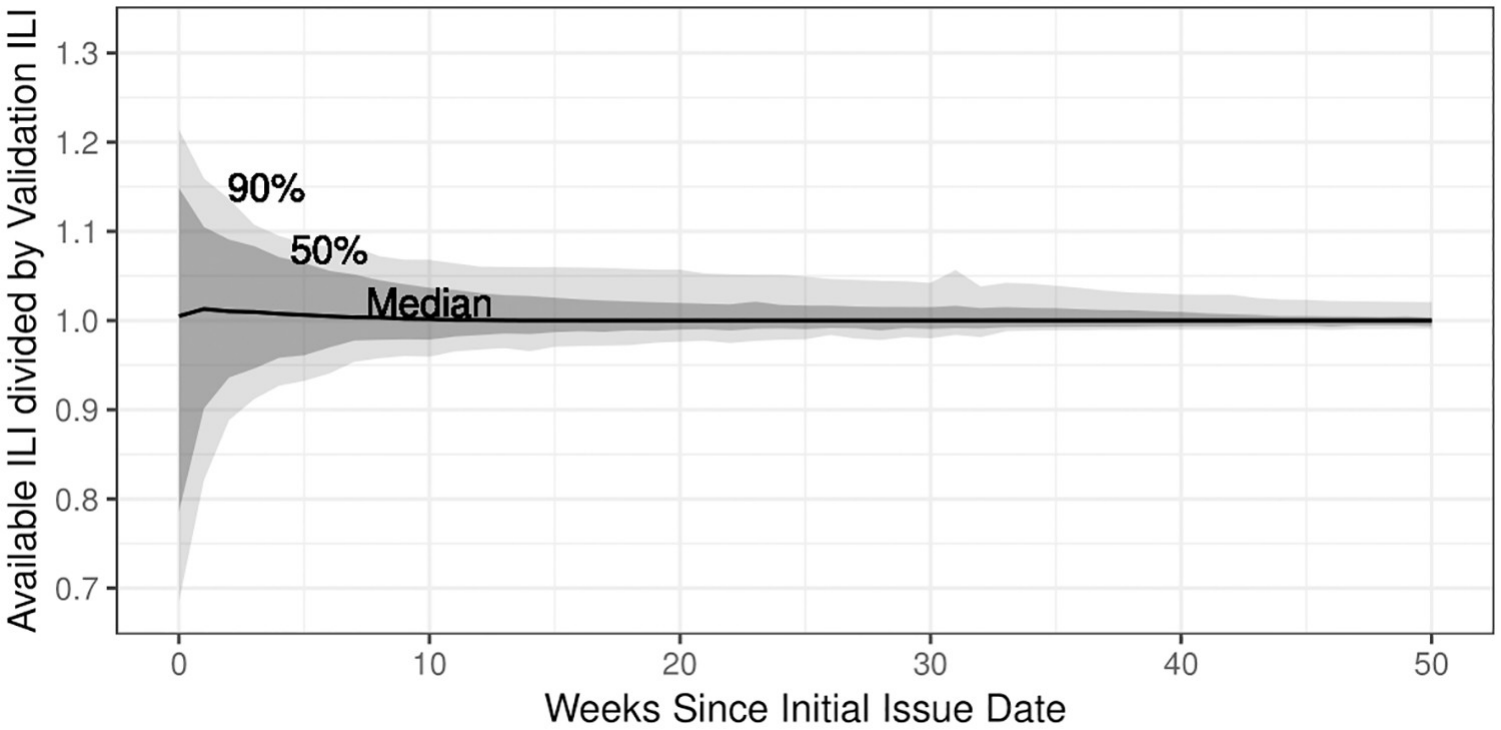
Changes to available ILI estimates as a function of weeks since the initial issue date, across all seasons and regions. 50% and 90% uncertainty intervals are shown. The effect of backfill diminishes as a function of weeks since the initial issue date and stabilizes after roughly 40 weeks.

Fig 2 shows the diminishing affect of backfill as a function of weeks since initial issue date.

- Initial ILI has historically been revised by up to ±25%.
- If 10 or more weeks have passed since the initial issue date, most ILI estimates have historically been revised by less than ±5%.
- If 40 or more weeks have passed since the initial issue date, most ILI estimates have historically been revised by less than ±2.5%.

**Fig 3:**
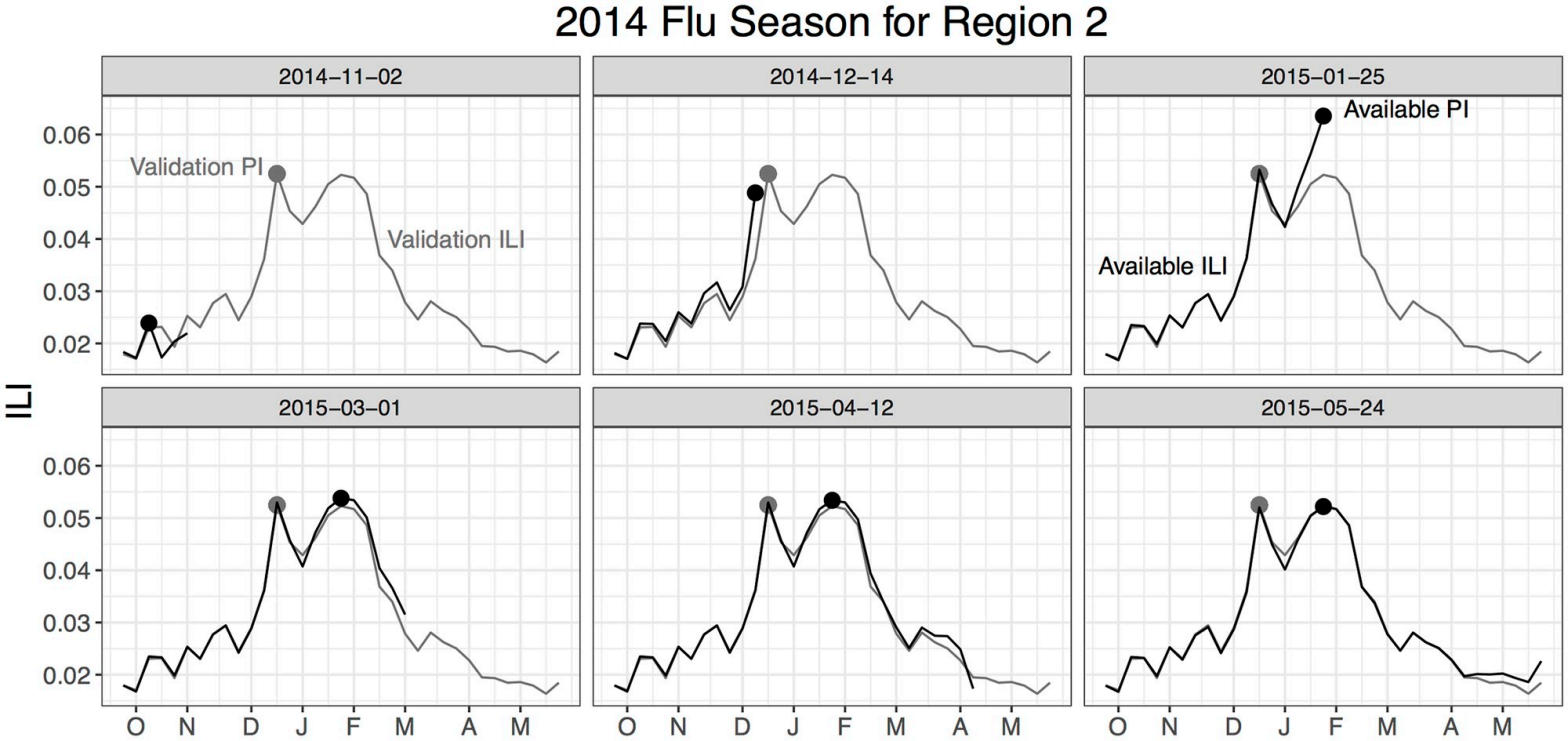
Backfill for the 2014 flu season for Region 2. The validation peak intensity is 0.0525 and occurs in the middle of December. The peak intensity based on ILI issued on May 24th, 2015 is 0.0522 and occurs at the end of January, a difference of 6 weeks even though the intensity of the two weeks changed only by 0.0003.

One of the issues with backfill is that even small modifications to ILI estimates can have large impacts on target evaluation. This is illustrated in Fig 3 for the 2014 flu season in Region 2. Using ILI issued on May 24th, 2015, the flu season peaked the week of January 25th, 2015 at 5.22%. Using the validation ILI, however, issued nearly three years later, the flu season peaked the week of December 21st, 2014 at 5.25%, a difference of six weeks. Even though the backfill from the end of May 2015 to March 2018 was minimal (0.03% for the peak intensity), the peak week shifted by over a month.

The existence of backfill demands a specific validation date. Based on Fig 2, the impact of backfill diminishes as a function of weeks since the initial issue date, suggesting a good validation date should be “a long time” after the latest initial issue date. For this work, the latest initial issue date is May 14th, 2017 and the validation date is March 2nd, 2018—a difference of nearly 42 weeks.

#### Wikipedia and Google Health Trends

We use Google Health Trends (GHT) web search volumes to nowcast the missing lag week of ILI data. This requires selecting search queries that are semantically related to influenza.

To do this, we used the Wikipedia inter-article link graph. A dataset [18] from our previous work [3] includes a list of 573 articles linked from “Influenza”, and our other previous work [19] mapped these articles to Google search query strings (i.e., what web users typed into the Google search box). Briefly, this mapping is a mechanical translation based on regular expressions. The only manual step beyond identification of the seed article (“Influenza”) is enumerating stop phrases such as “and”, “list of”, and “influenza virus subtype”.

We used the Google Health Trends API [20] to download the weekly search volume for the 573 queries over the study period for the U.S. nationally and each state. This was done in batches from February 16 to March 5, 2018. The GHT API returns summaries of search activity based on a random sample and thus cannot be deterministically reproduced.

### Methods

In the following sections, we describe the model used for flu forecasting, the internet-based nowcasting procedure, the experimental setup, and the procedure for assessing the experiment.

#### Dynamic Bayesian Model

**Fig 4:**
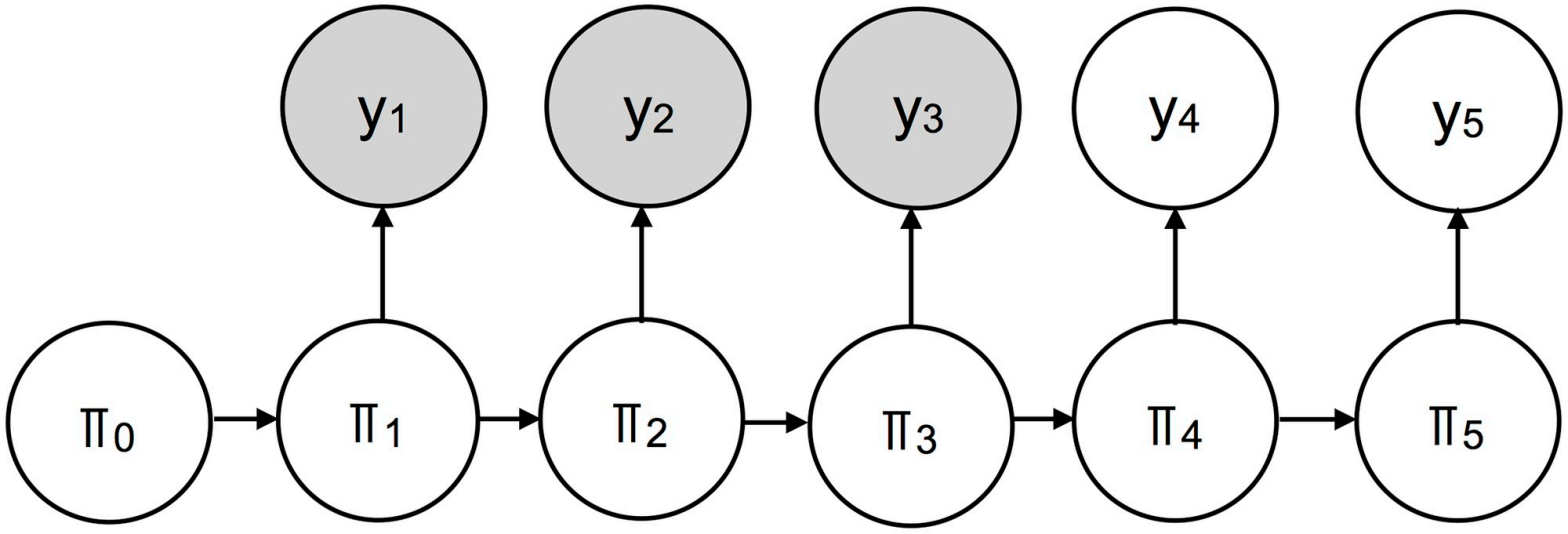
A simplified graphical representation of DBM. *π*_*t*_ represents latent ILI states, while *y*_*t*_ represents available ILI data. Shaded nodes represent observed quantities; unshaded nodes represent unobserved quantities. Directed edges represent dependencies between nodes.

The Dynamic Bayesian Model (DBM) is a hierarchical Bayesian model. A simplified, graphical representation of DBM is shown in Fig 4. The quantity *π*_*t*_ represents a latent state of ILI transmission on week *t*, linked in time through directed edges. Observations of available ILI (*y*_*t*_) are linked to latent ILI states. For a given forecast date, some of the available ILI nodes are observed (shaded) while others are unobserved (unshaded). DBM is a statistical model that estimates the unobserved nodes, given the observed nodes. As the flu season progresses, more available ILI nodes are observed and estimation of the shrinking set of unobserved nodes is refined.

The full description of DBM is somewhat involved; the interested reader is directed to [16] for more details than are presented here. For the purposes of this paper, it suffices to know that DBM is a hybrid model, combining the structure of a compartmental disease transmission model with the flexibility of a statistical model.

Forecasting with DBM is performed by sampling from the *posterior predictive distribution*—the distribution of unobserved available ILI nodes given observed available ILI nodes—via Markov chain Monte Carlo (MCMC). The MCMC sampling is done using the rjags package [21] within R [22]. In this paper, all results are based on runs of three chains of 15,000 iterations per chain with a burn-in of 5,000 iterations and thinning every 5th iteration. Thus, summaries of the posterior predictive distribution are based on 6,000 samples. Multiple chains were run to assess convergence to the stationary distribution, 15,000 iterations per chain were selected for chain convergence, and thinning every 5th iteration was chosen to address chain mixing.

The posterior predictive distribution does not explicitly capture the effect of backfill. Thus, a modification is applied to each draw (*j*) from the posterior predictive distribution ($y_{jt}^{*}$) and the available ILI (*y*_*t*_) on week *t* as follows:

$$\begin{array}{cc} {\tilde{y}}_{jt} & =\{\begin{array}{cc} {\left(\kappa /n_{t}\right)}^{2}y_{jt}^{*}+\left(1-{\left(\kappa /n_{t}\right)}^{2}\right)y_{t} & \;\text{if}\;y_{t}\;\text{is}\;\text{observed}\\ y_{jt}^{*} & \;\text{if}\;y_{t}\;\text{is}\;\text{unobserved,} \end{array} \end{array}$$ (1)

where *ỹ*_*jt*_ is a modified estimate of validation ILI accounting for backfill on week *t*, *n*_*t*_ ≥ 1 is the number of weeks since the initial issue date of *y*_*t*_, and *κ* is a tuning parameter that controls the weight of the convex combination of $y_{jt}^{*}$ and *y*_*t*_. Eq 1 is motivated by Fig 2. Available ILI becomes a better estimate of validation ILI as the number of weeks since the initial issue date increases. Eq 1 places more weight on available ILI *y*_*t*_ as the number of weeks since the initial issue date *n*_*t*_ increases, capturing the diminishing affect of backfill illustrated in Fig 2. The parameter *κ* was set at 0.75—a pragmatic choice. Alternatively, *κ* could have been chosen more formally via cross-validation.

**Fig 5:**
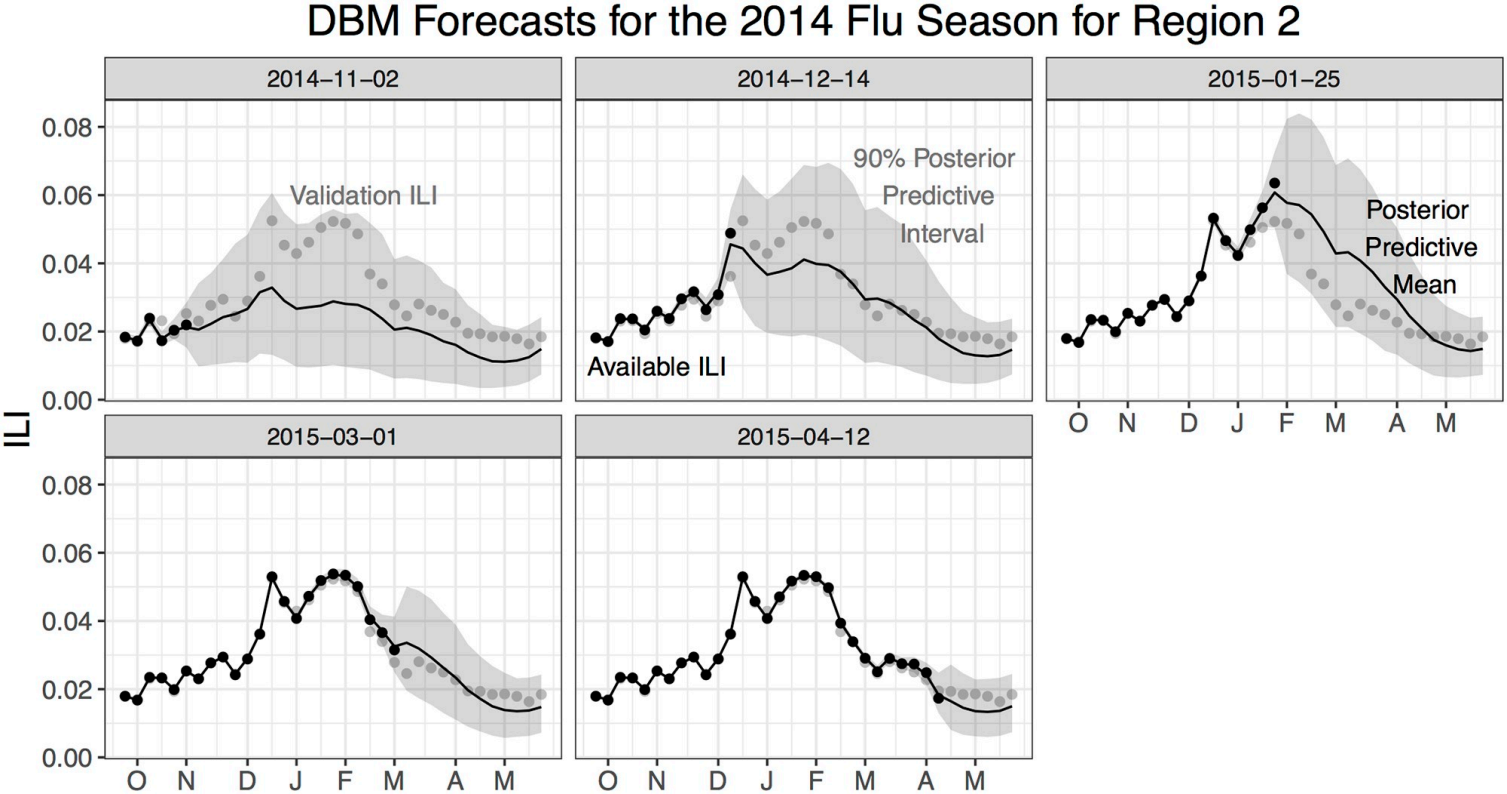
DBM forecasts for validation ILI for the 2014 flu season for Region 2. Posterior predictive summaries of *ỹ*_*jt*_ are presented, based on the available ILI for each panel. Notice there is forecast uncertainty even for weeks where available ILI exists, accounting for backfill.

Fig 5 shows summaries of the backfill-modified forecasts (*ỹ*_*jt*_) for Region 2 for the 2014 flu season. We see as more available ILI is incorporated into DBM fitting, forecasts are updated. Note that there is forecast uncertainty for weeks where available ILI is observed. That uncertainty accounts for backfill. The degree of forecast uncertainty around available ILI diminishes as the number of weeks since the initial issue date increases.

DBM does a good job capturing the validation ILI, with respect to its predictive uncertainty, for all forecasts throughout the season. In fact, [16] found that, when comparing DBM forecasts nationally to all models that participated in the CDC’s 2015/16 and 2016/17 flu forecasting challenges, DBM outperformed all competing models; i.e., DBM is a leading forecasting model. It is worth mentioning that DBM only uses ILI estimates as a data source; it does not make use of any external data, such as internet-based information. In subsequent sections, we discuss how DBM, an already-good forecasting model, can be augmented to incorporate internet-based information and assess the value of that augmentation.

#### Internet-based nowcasting

The purpose of ILI nowcasting is to fill the gap created by the CDC’s ILI reporting lag. In this paper, we treat the reporting lag as one week. Our approach, however, can trivially be extended to a *k*-week reporting lag, with *k* ≥ 1.

A good nowcast is one that predicts the yet-to-be-issued one-week-ahead validation ILI. We use LASSO regression for ILI nowcasting. It is important to note that our objective in this section is not to identify the “best” model for nowcasting. Rather it is to 1) identify a “good” model for nowcasting and 2) understand the limits on forecasting improvement that nowcasting provides, independent of the selected nowcasting model. That said, the remainder of the section provides evidence in support of our decision to nowcast with LASSO regression.

The space of nowcasting approaches presented in the literature is large, including time series autoregression [23], regularized regression [24], support vector machines [11], neural nets [25], and random forests [26]. We consider elastic net regression models, a form of regularized regression, for nowcasting. Elastic net regression solves the following minimization:

$$\hat{\beta }=\underset{\beta }{\text{arg}\;\text{min}}\sum_{i=1}^{N}(y_{i}-\beta_{0}-\sum_{p=1}^{P}X_{i,p}\beta_{p}{)}^{2}+\lambda (\frac{1-\alpha }{2}\sum_{p=1}^{P}\beta_{p}^{2}+\alpha \sum_{p=1}^{P}|\beta_{p}|),$$ (2)

where λ ≥ 0 and *α* ∈ [0, 1] are tuning parameters [27]. When *α* = 1, elastic net regression reduces to LASSO regression with penalty parameter λ. When *α* = 0, elastic net regression reduces to ridge regression with penalty parameter λ/2. We use the glmnet package in R to carry out the elastic net parameter estimation [28].

We considered elastic net nowcasting models corresponding to *α* = 0, 0.1, 0.2, …, 1. The following procedure was used to produce one nowcast for each region/season/forecast week. The procedure was repeated for each region/ season/forecast week.

1. Let ***X***_*j*,*t*_ be the *N*_*j*,*t*_ × (*P* + 1) training matrix. Each non-intercept column corresponds to one of the *P* = 573 GHT search queries and each row corresponds to a week. *N*_*j*,*t*_ includes data from flu seasons *j* − 2 and *j* − 1, as well as the first *t* weeks of flu season *j*. Thus, the nowcasting model is always trained on between two and three full flu seasons, inclusive.
  - When nowcasting for a HHS region (as opposed to nationally), $X_{j,t}^{\text{state}}$ is constructed for each state in the HHS region. $X_{j,t}^{\text{region}}$ is then constructed by taking a population-weighted average of training matrices, where the state weights are proportional to their 2010 Census population estimates.
2. Let ***Y***_*j*,*t*_ be the *N*_*j*,*t*_ × 1 vector of available ILI estimates issued on week *t* of flu season *j* corresponding to the weeks of ***X***_*j*,*t*_.
3. For each value of *α*, fit elastic net regression, using 10-fold cross-validation for the selection of λ that minimizes the cross-validated root mean-squared error (RMSE).
4. Given the fitted elastic net model and ***X***_*j*,*t*+1_ (the GHT search query activity for the nowcasting week), predict *ÿ*_*j*,*t*+1_, the validation ILI for week *t* + 1. Call the nowcast *ŷ*_*j*,*t*+1_.

For each nowcasting model, we computed summaries of the correlation and RMSE across all 55 region/season pairs. Results are shown in Table 1. We see that ridge regression (*α* = 0) produces qualitatively worse nowcasts than all other considered elastic net regressions, suggesting that some amount of *ℓ*_1_ penalty on the regression coefficients improves nowcasting. The *ℓ*_1_ penalty is the mechanism by which elastic net regression can “zero out” either unhelpful and/or redundant features. In general, the average number of estimated non-zero coefficients reduces as *α* increases, with a steep drop between *α* = 0 and *α* = 0.1. Elastic net for *α* ≥ 0.1 results in correlations, averaged over all regions and seasons, of ∼ 0.88 and RMSEs of ∼ 0.005. There is little difference between the average estimated correlations and RMSEs, as well as their estimated ranges, for *α* ≥ 0.1. For these reasons, we use LASSO regression (i.e., *α* = 1) for nowcasting.

**Table 1:** Nowcast performance for elastic net models of *α* = 0.0, 0.1, …, 1.0. The correlation is the average correlation over all 55 region/season pairs, while the min and max correlations are the min and max correlations over all region/season pairs. Similarly for the RMSE. The average number of estimated non-zero features are shown in the “Features” column. Ridge regression is qualitatively worse than all other *α* > 0 models. There is little difference between all *α* ≥ 0.1 models with respect to correlation and RMSE. The number of non-zero features tends to decline as an increasing function of *α*.

*α*	Cor (min, max)	RMSE (min, max)	Features
0.0 (ridge)	0.775 (0.113, 0.971)	0.0078 (0.0025, 0.0220)	357
0.1	0.872 (0.522, 0.981)	0.0052 (0.0022, 0.0134)	51
0.2	0.878 (0.553, 0.982)	0.0052 (0.0022, 0.0134)	35
0.3	0.881 (0.580, 0.982)	0.0051 (0.0022, 0.0135)	28
0.4	0.884 (0.566, 0.983)	0.0051 (0.0022, 0.0136)	25
0.5	0.884 (0.594, 0.985)	0.0051 (0.0021, 0.0138)	23
0.6	0.884 (0.593, 0.986)	0.0051 (0.0021, 0.0138)	22
0.7	0.884 (0.582, 0.986)	0.0052 (0.0021, 0.0138)	20
0.8	0.881 (0.576, 0.988)	0.0052 (0.0020, 0.0139)	20
0.9	0.881 (0.584, 0.988)	0.0052 (0.0020, 0.0139)	19
1.0 (LASSO)	0.885 (0.575, 0.988)	0.0052 (0.0019, 0.0138)	17

#### Experimental setup

**Fig 6:**
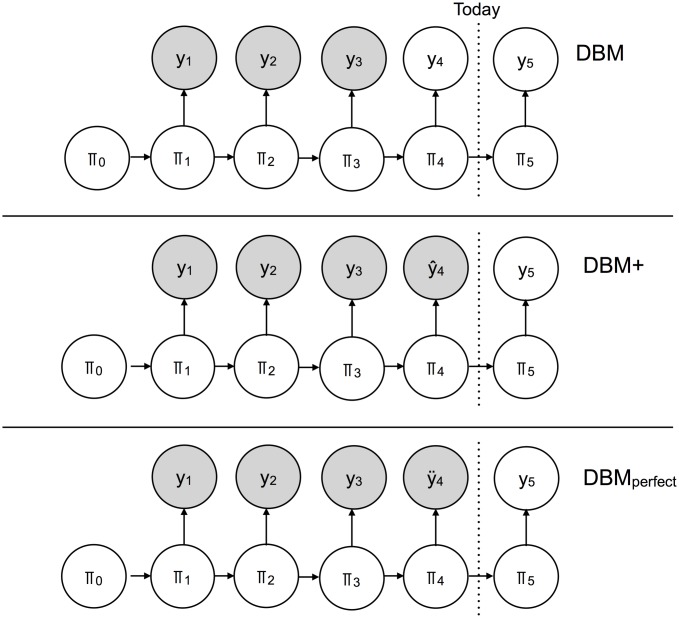
The simplified graphical representations of DBM, DBM+, and DBMperfect. *π*_*t*_ is the latent state of ILI for week *t*, while *y*_*t*_ is available ILI. Observed nodes are shaded; unobserved nodes are unshaded. The ILI reporting lag creates a gap between the day a forecast is rendered (vertical “Today” line) and the most recent available ILI estimate (*y*_3_). DBM does not fill in the reporting gap, denoted by the unshaded node *y*_4_. DBM+ fills in the reporting gap with a nowcast, denoted by the shaded node *ŷ*_4_. DBMperfect cheats by filling the reporting gap with the actual validation ILI, denoted by the shaded node *ÿ*_4_.

Our goal in this paper is to interrogate the value of internet-based nowcasts when incorporated into a good internet-free forecasting model. To do so, we conduct an experiment where we compare three statistical models across various scenarios. The three statistical models are illustrated in Fig 6.

The first model is DBM. Due to the ILI reporting lag, there is a one week gap between the most readily available ILI estimates and the forecast date. DBM does not fill in that gap with a nowcast.

The second model is DBM+. DBM+ is the same as DBM, except DBM+ fills in the ILI gap with an internet-based nowcast. For DBM+, the nowcast is treated just like a CDC-issued ILI estimate. Based on Table 1, the nowcasts are relatively highly correlated with the validation ILI and have an average RMSE of ∼ 0.005. That being said, the nowcasts do not perfectly match the validation ILI they are estimating.

The third model we consider is DBMperfect. DBMperfect cheats by filling in the ILI gap with the actual validation ILI estimate. Unlike DBM and DBM+, DBMperfect cannot be used for forecasting in real-time, as the validation ILI estimates required to run DBMperfect are not available when forecasts are rendered. DBMperfect, however, serves as an upper bound on the possible DBM improvement one can expect when using any nowcast. How well DBM+ approximates the performance of DBMperfect is directly related to the quality of the nowcasts.

The experiment we conduct compares the forecasting performance of DBM, DBM+, and DBMperfect across three factors:

1. **Geography (11 levels)**: Models are fit at the national level as well as the 10 HHS regions (data shown in Fig 1).
2. **Flu Seasons (5)**: Models are fit to the 2012 through the 2016 flu seasons.
3. **Epidemic Weeks (25)**: Models are fit for 25 consecutive weeks, starting on epidemic week 44, roughly the end of October, and ending roughly at the middle-to-end of April.

Thus, each model renders forecasts for 11 × 5 × 25 = 1375 scenarios.

#### Experimental assessment

We assess the models based on their ability to forecast seven targets specified by the CDC’s flu forecasting challenge [29]:

- **Onset**: the first of three consecutive weeks equal to or above baseline, where baseline is a region/season-specific quantity determined by the CDC
- **Peak intensity (PI)**: the maximum validation ILI estimate for the flu season
- **Peak timing (PT)**: the week(s) the maximum validation ILI estimate for the flu season is observed
- **Short-term forecasts**: one- through four-week-ahead forecasts, where week-ahead forecasts are with respect to available ILI. As a concrete example, in Fig 6, a one-week-ahead forecasts for all models means forecasting week 4, as the last available ILI week is week 3.

Forecasts for each target are in the form of a probability distribution, capturing the uncertainty in the target-specific forecasts. Accuracy is assessed with a modified log scoring rule following that of the CDC’s flu forecasting challenge [29]. Let

$$p_{m,r,s,\tau ,t}=(p_{m,r,s,\tau ,t,1},p_{m,r,s,\tau ,t,2},\ldots ,p_{m,r,s,\tau ,t,n_{\tau }}{)}^{\prime }$$ (3)

be a length *n*_*τ*_ vector of probabilities corresponding to bins for model *m*, region *r*, flu season *s*, target *τ*, and issue week *t*, such that all elements of ***p***_*m*,*r*,*s*,*τ*,*t*_ are non-negative and $\sum_{i=1}^{n_{\tau }}p_{m,r,s,\tau ,t,i}=1$. For targets onset and PT, bins correspond to weeks. For all other targets, bins are equally spaced intervals of width 0.001 from 0 to 0.13, with a final bin equal to (0.13, 1.00].

For a target, let the correct value as determined by the validation ILI be equal to bin *ĩ*. Then, the modified log score *l*_*m*,*r*,*s*,*τ*,*t*_ is computed as

$$l_{m,r,s,\tau ,t}=\text{max}(-10,\text{ln}(\sum_{j\in \tilde{I}}p_{m,r,s,\tau ,t,j})),$$ (4)

where

$$\tilde{I}=\{\tilde{i}-1,\tilde{i},\tilde{i}+1\},$$ (5)

for onset and PT and

$$\tilde{I}=\{\tilde{i}-.005,\tilde{i}-.004,\ldots ,\tilde{i}+.004,\tilde{i}+.005\},$$ (6)

for PI and the short-term forecasts, and ln() is the natural log. If, for instance, the peak timing occurred on epidemic week 5 (*ĩ* = 5), then *Ĩ* = {4, 5, 6}. If the forecast assigned probability 0.05, 0.2, and 0.15 to the epidemic weeks of *Ĩ*, respectively, then the modified log score would be ln(0.05 + 0.2 + 0.15) = −0.92.

Note that *l*_*m*,*r*,*s*,*τ*,*t*_ ∈ [−10, 0] and is not particularly interpretable. We employ a simple, monotonic transformation to the modified log score,

$$s_{m,r,s,\tau ,t}=\text{exp}(l_{m,r,s,\tau ,t}),$$ (7)

referred to as the *forecast skill*, where *s*_*m*,*r*,*s*,*τ*,*t*_ ∈ [0, 1] (or more specifically, [exp(−10), 1]) and exp() is the exponential function.

Throughout this work, we compute summaries of the modified log score and, hence, summaries of the forecast skill, where we average over indices, denoted by “·”. For instance, the model-specific average modified log score is denoted as *l*_*m*,.,.,.,._ and is the average modified log score, averaged over all regions, seasons, targets, and issue weeks. The corresponding model-specific average forecast skill is *s*_*m*,.,.,.,_ = exp(*l*_*m*,.,.,.,._). The model-specific average forecast skill then has the interpretation of the geometric mean of the model-specific average modified log score. For reference, the winning forecast skill for the CDC’s 2016/17 challenge was *s*_*m*,.,2016,.,._ ≈ 0.45.

## Results

**Fig 7:**
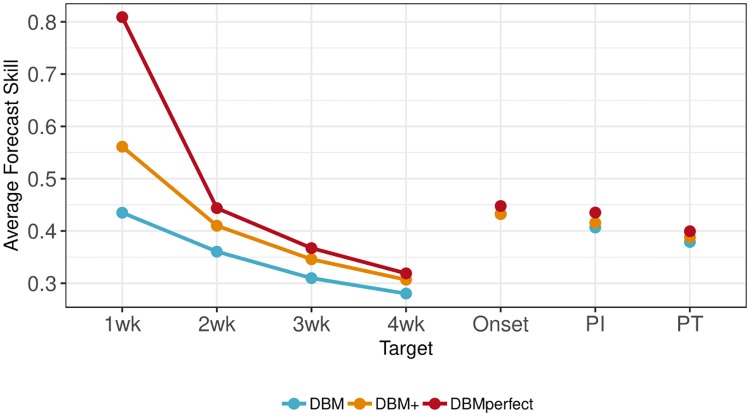
Target-specific average forecast skill (*s*_*m*,.,.,*τ*,._) averaged across all regions, seasons, and weeks. DBMperfect has the best forecast skill for all short-term forecasts, followed by DBM+, and finally DBM, respectively. The largest improvement in forecast skill relative to DBM occurs for one-week-ahead forecasts (i.e., nowcasts), as expected, as DBMperfect has perfect knowledge of nowcasts (but *s*_DBMperfect,.,.,*τ*,._ ≠ 1 because of backfill), while DBM+ has an external estimate of the nowcast. All models exhibit similar forecast skills for seasonal targets (i.e., onset, PI, and PT), with a slight improvement for DBMperfect over DBM for all three seasonal targets.

Fig 7 displays the average forecast skill over all seasons, regions, and issue weeks for each target and model. The average forecast skill, smallest to largest, is DBM, DBM+, and DBMperfect for each target, indicating that DBM’s forecasting ability can be improved by including internet-based nowcasts in the manner outlined above. Further, DBMperfect provides bounds on the degree of improvement that can be achieved through nowcasting.

Not surprisingly, nowcasts provide the greatest potential for forecast improvement of the one-week-ahead target. This is because the nowcast provides a direct estimate of the one-week-ahead target. The reason the forecast skill is not 1 for DBMperfect’s one-week-ahead forecasts is backfill. Though we imputed the validation ILI for the nowcast, DBMperfect does not know the nowcast is perfect. The backfill component of DBM still runs (i.e., Eq 1), resulting in one-week-ahead forecasts for DBMperfect with uncertainty and hence, forecast skills not equal to 1. Because, however, DBM’s modeling components are all connected (see Fig 6), direct information for the one-week-ahead target has rippling effects to all other targets. We see that two- through four-week-ahead forecasts are also uniformly improved when comparing DBM’s average forecast skill to DBM+ and DBMperfect, though both the magnitude of improvement and magnitude of overall forecast skill declines with increased lead times. The seasonal targets (i.e., onset, PI, and PT) are improved by a relatively small amount when internet-based nowcasts are used, but improved nonetheless. The small difference between DBM and DBM+ forecast skill for seasonal targets relative to short-term targets is not wholly surprising, as nowcasts do not provide direct estimates of these targets. However, the fact that forecast skill did uniformly improve for seasonal targets when compared to DBM is encouraging, as the incorporation of nowcasts into DBM’s modeling framework can improve all aspects of forecasting, not just short-term forecasts.

**Fig 8:**
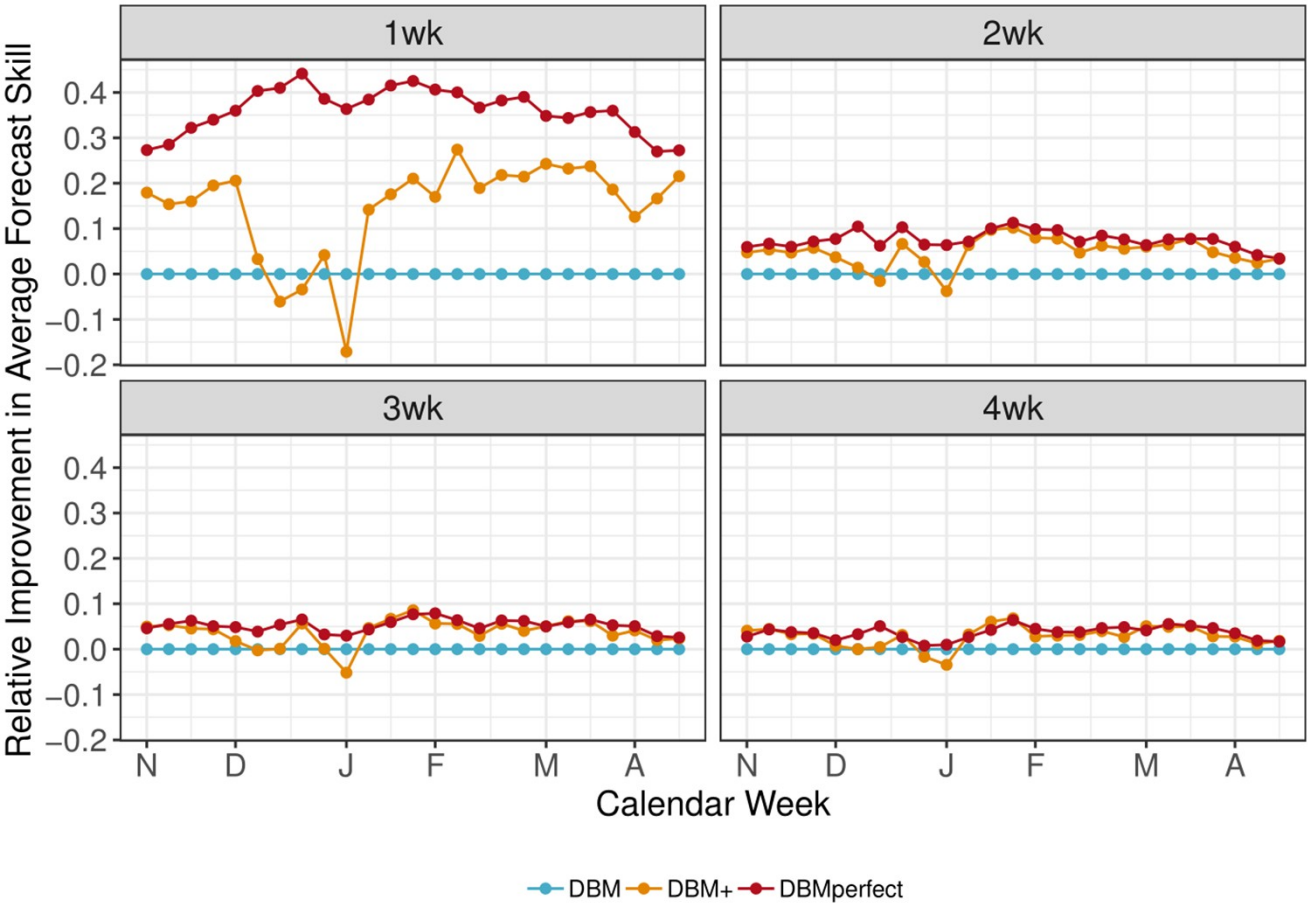
Target and week-specific relative average forecast skill (*s*_*m*,.,.,*τ*,*t*_ − *s*_DBM,.,.,*τ*,*t*_) averaged across all regions and seasons. DBMperfect has better average forecast skill than DBM for all weeks and short-term targets. DBM+ has better average forecast skill than DBM most of the season, but tends to have worse average forecast skill during December and early January—the time of year ILI tends to rapidly increase.

When average forecast skill is considered on a weekly basis, however, we see there are weeks of the season when DBM+ has worse skill than DBM. Fig 8 shows the average forecast skill improvement relative to DBM for DBM+ and DBMperfect. DBM+ has better average forecast skill for most weeks of the season for all short-term targets, but actually can have worse average forecasting skill during December and early January—the time of year when ILI tends to rapidly increase. This suggests that nowcasts used in DBM+ provide biased information and DBM+ would, in fact, be better off using no nowcasts than the ones generated by LASSO during December and early January. Averaged over the whole season, however, the LASSO nowcasts used by DBM+ yield better short-term forecasts than DBM. Fig 8 makes clear the most substantial opportunity for nowcasting improvement is during December and January. In contrast, DBMperfect has better average forecast skill than DBM for all weeks of the season and all short-term forecasting targets, indicating that DBM’s forecast skill can be improved uniformly if nowcasting can be done perfectly.

**Fig 9:**
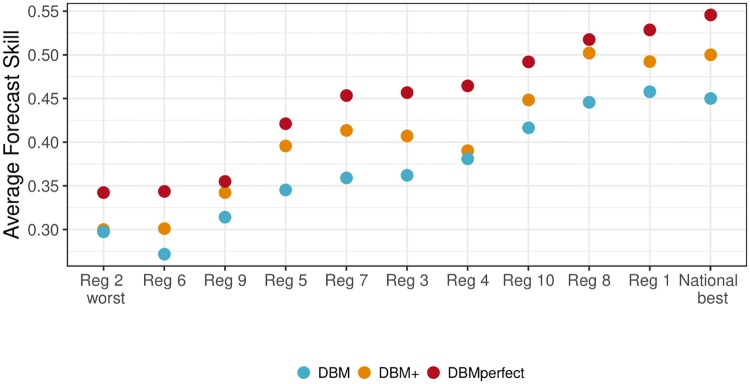
Average forecast skill by region, averaged over all seasons and targets (*s*_*m*,*r*,.,.,._). Regions are ordered by increasing DBMperfect average forecast skill. For all regions, DBM has the lowest average forecast skill, followed by DBM+ and DBMperfect, respectively.

Fig 9 plots the region-specific average forecast skills (*s*_*m*,*r*,.,.,._) for all regions. The overall forecast skill varies by region, with the lowest forecast skill for Region 2 and highest forecast skill nationally. For every region, however, we see DBM+ has better forecast skill than DBM, while DBMperfect has better forecast skill than DBM+. The improvement of DBM+ over DBM varies; for some regions the improvement is relatively small (e.g., Regions 2 and 4), while in other regions it approaches that of DBMperfect (e.g., Regions 8 and 9). For all regions, though, DBM+ improves forecast skill over DBM, an appealing result.

**Fig 10:**
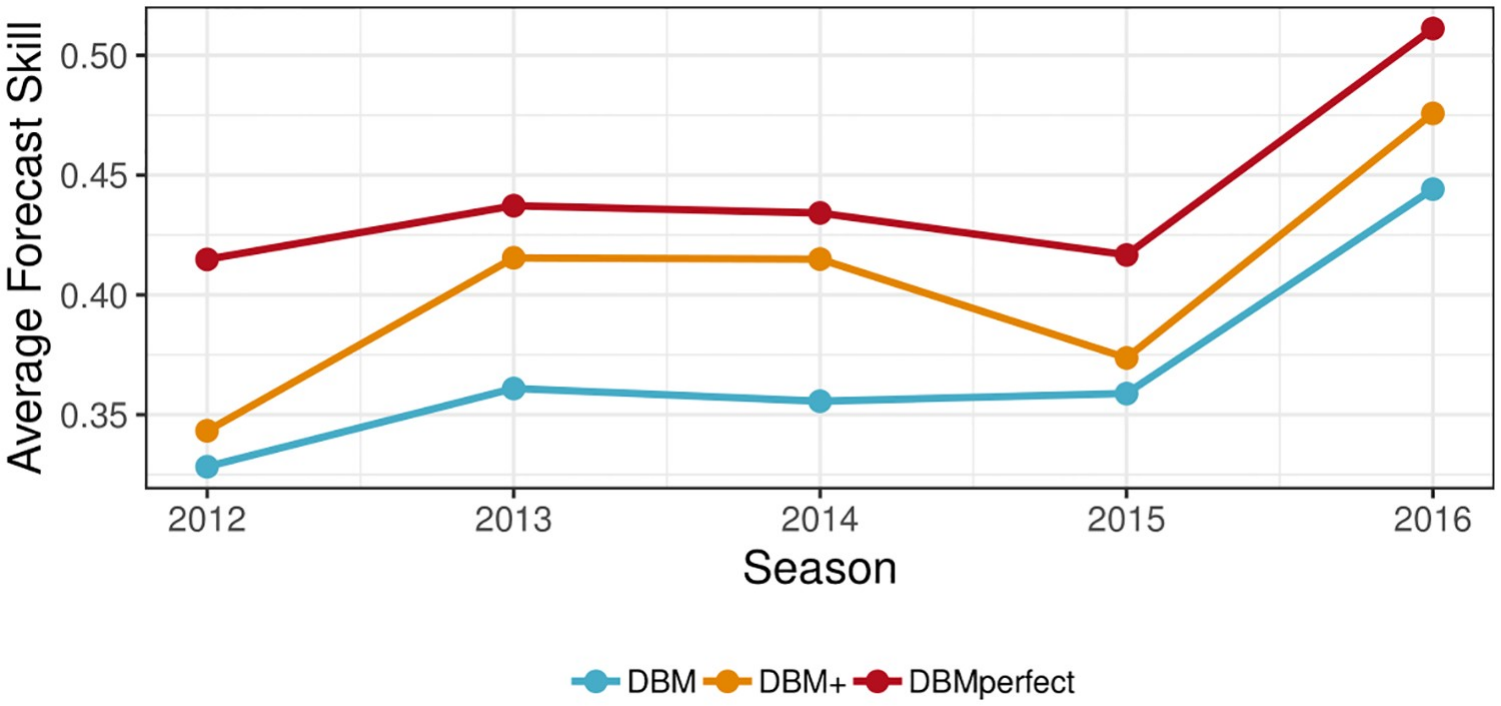
Average forecast skill by season, averaged over all regions and targets (*s*_*m*,.,*s*,.,._). DBMperfect has the highest average forecast skill for all seasons, followed by DBM+ and DBM, respectively.

Finally, we compute the season-specific average forecast skill (*s*_*m*,.,*s*,.,._), displayed in Fig 10. Here we see that average forecast skill varies from season-to-season, with the lowest forecast skill for 2012 and the highest for 2016. For all seasons, DBMperfect has the best forecast skill, followed by DBM+ and finally DBM. The improvement of DBM+ relative to DBM varies across seasons. In some years, the improvement is relatively small (e.g., 2012 and 2015) while in others it is larger (e.g., 2013 and 2014). For all seasons, though, DBM+ improves the forecast skill over DBM.

Figs 7, 8, 9 and 10 provide strong evidence that DBM can be improved through the inclusion of internet-based nowcasts. These conclusions hold across all considered targets, regions, seasons, and most weeks of the season.

The improvement of DBM+ over DBM does not just exist, however, it is also appreciable. The model-specific average forecast skills, averaged over all regions, season, targets, and issue weeks are

$$\begin{array}{r} s_{\text{DBM},.,.,.,.}=0.368,\\ s_{\text{DBM+},.,.,.,.}=0.402,\\ s_{\text{DBMperfect},.,.,.,.}=0.442. \end{array}$$

The difference between *s*_DBM+,.,.,.,._ and *s*_DBM,.,.,.,._ is 0.034. While seemingly small, that is roughly a difference between 1st place and 7th place in the CDC’s 2016/17 30-model flu forecasting challenge.

The upper bound on forecast skill improvement is captured by the difference in average forecast skill between *s*_DBMperfect,.,.,.,._ and *s*_DBM,.,.,.,._. That difference is 0.074, roughly the difference between 1st place and 11th place. An improvement equivalent to a 7th to 1st place jump in practice, with the potential for an 11th to 1st place jump simply by incorporating internet-based nowcasts represents an appreciably large improvement.

Finally, the difference between DBMperfect and DBM+ represents the magnitude of improvement left unexploited by our LASSO nowcasting model. This difference is 0.04—larger than the improvement of DBM+ over DBM. Thus, though DBM+ provides significant gains over DBM, there is still potential for significant improvements to DBM+ via improved nowcasting. Fig 8 highlights that improvements to nowcasting during December and early January are good places to start. If, however, improvements above and beyond what DBMperfect provide are desired, improving the forecasting model through nowcasting will be insufficient, as DBMperfect provides an upper bound on the improvement one can expect from nowcasting, *regardless of nowcasting modeling and data choices*.

## Discussion

We performed a focused experiment to provide clarity on the value of internet data when forecasting the flu in practice, designed to provide answers to specific questions:

1. **Can a good flu forecasting model using no internet data be improved by adding internet data?**
  Yes. The experiment demonstrated that DBM can be improved in practice by adding internet-based nowcasts, as was demonstrated by DBM+. DBM+ had equal or better forecast skill for all considered forecasting targets, averaged over all seasons and regions, and had better overall forecasting skill (0.402) than did DBM (0.368); an improvement comparable to the difference between 7th and 1st place in the CDC’s 2016/17 flu forecasting challenge.
2. **If so, do the improvements hold across flu targets, season weeks, geographic regions, and flu seasons?**
  Generally yes. When comparing the average forecast skill of DBM+ to DBM, DBM+ had equal or better forecast skill for all flu targets (Fig 7), geographic regions (Fig 9), and flu seasons (Fig 10). The magnitude of improvement of DBM+ to DBM varied by target, region and season, but in all instances, DBM+ had equal or better forecast skill than DBM. DBM+, however, yielded worse average forecast skill for short-term forecasting targets made in December and early January (Fig 8), pointing to an opportunity for nowcasting improvement.
3. **What are the limitations of nowcasts, internet-based or otherwise, in terms of improved forecasting performance?**
  DBMperfect’s forecast skill provides an upper bound on the possible improvements for DBM when augmented with nowcasts. Those improvements are appreciably large, with improvements comparable to the difference between the 1st and 11th place models in the CDC’s 2016/17 flu forecasting competition. However, DBMperfect makes explicit that the possible improvements to DBM via even perfect nowcasting, though substantial, are uneven and limited. Short-term forecasting targets see the largest improvement, but forecasting improvements diminish as the forecast window increases. Average forecast skill for seasonal targets improve, but the improvement relative to short-term forecasting targets is small. Thus, larger improvements to seasonal target forecast skill will need to be derived from other approaches.

The experiment described in this paper provides clear and concise evidence for several questions relevant to the field of flu forecasting, which is in its infancy and ripe for improvement. Much effort has been spent focusing on improvements to forecasting via the augmentation of forecasting models with internet-based nowcasts. This paper provides clear evidence that 1) forecasting, in practice, can be substantially improved with internet-based nowcasts and 2) improvements based on nowcasts, internet-based or otherwise, are limited. For instance, if a flu forecasting model was needed to achieve an average forecast skill of 0.5, augmenting DBM with nowcasts, even perfect nowcasts, will likely fall short. That is, even augmenting DBM with perfect nowcasts does not achieve the needed average forecast skill, making it clear that improvements to DBM above and beyond DBMperfect will need to come from other, non-nowcasting approaches, such as multiscale modeling, spatial modeling, ensemble modeling, and/or the incorporation of additional, available data streams such as flu strain and/or vaccination data.

There are many questions related to this work worthy of further investigation that are outside the scope of this paper. Some include:

- **Do these findings extend to other non-DBM flu forecasting models?** The conclusions we draw are DBM-specific. Extending them beyond DBM would require similar experiments for different flu forecasting models. That said, we speculate that our findings are likely to hold for most if not all flu forecasting models. For instance, our finding that forecast skill for seasonal targets only marginally improves relative to short-term forecasting targets, even with perfect nowcasting, seems likely to hold across a spectrum of flu forecasting models. This is because a nowcast is a direct estimate of a one-week-ahead forecast and is only indirectly related to all other targets. It is hard to imagine, for instance, a model where perfect knowledge of a one-week-ahead forecast is also highly informative of the peak intensity of the flu season.
- **What happens if we increase the nowcasting window?** One simple extension of this work is to consider nowcasting as more than a one-week-ahead forecast. If, for instance, nowcasting incorporated one- and two-week-ahead forecasts, improvements to overall forecast accuracy would likely improve relative to the results presented here.
- **What are the drivers of backfill? Can we predict backfill?** Backfill is a consequence of a mature, real-world data collection system (e.g., ILINet). From a forecasting perspective, it is important to account for backfill for the purposes of reproducibility as well as faithful comparisons to other models. From a practical perspective, backfill makes decision making in real-time challenging as the data available at any moment may or may not provide an adequate picture of disease activity. Consider Fig 3 and the decision whether to allocate additional resources to Region 2 based on the data available on January 25th, 2015. Based on the large increase in available ILI activity, a decision maker might choose to allocate additional resources. If, however, the validation ILI were available in real-time, the decision maker may have decided against the additional allocation. Considering backfill must be part of the decision-making process. A better understanding of the main drivers of backfill and ultimately a model to accurately predict its behavior is needed but currently lacking.
- **What makes some regions and seasons harder to forecast than others?** Figs 9 and 10 illustrate heterogeneity in DBM forecasting skill across regions and seasons. The reasons for this have not been thoroughly investigated. It could be that DBM intrinsically struggles when forecasting higher flu-like illness activity regions, as the lower forecast skill regions (e.g., Regions 2, 6, and 9) also have the highest average ILI. Week-to-week ILI variability, however, is correlated with average ILI. Thus, it could be the volatile week-to-week changes in ILI is what makes forecasting some regions hard, and the fact they are also high average ILI regions is purely incidental. Considering what makes seasons challenging to forecast is difficult based on the presented analysis as only five flu seasons were considered. In our previous work [16], we found that DBM forecast quality was related to how “similar” a flu season was to previously seen flu seasons, as DBM models all available flu seasons within a region jointly. This finding, however, was only considered at the national scale. A thorough investigation into what makes regions and seasons more challenging to forecast than others is warranted. Understanding the drivers of heterogenous forecast skill would provide useful insights into how to improve forecasting models in the future and articulate potential forecasting vulnerabilities.

## Supporting information

S1 Dataset Forecast skill scores for DBM, DBM+, and DBMperfect. (CSV)

S2 Dataset Google Health Trends terms used for nowcasting and the number of times each was assigned a non-zero coefficient via LASSO regression. (CSV)

S3 Dataset One-week-ahead nowcasts from LASSO regression. (CSV)
